# A Dairy Product to Reconstitute Enriched with Bioactive Nutrients Stops Bone Loss in High-Risk Menopausal Women without Pharmacological Treatment

**DOI:** 10.3390/nu12082203

**Published:** 2020-07-24

**Authors:** Marina Morato-Martínez, Bricia López-Plaza, Cristina Santurino, Samara Palma-Milla, Carmen Gómez-Candela

**Affiliations:** 1 Nutrition Department, La Paz University Hospital Institute for Health Research (IdiPAZ), Autonomous University of Madrid, 28046 Madrid, Spain; marina.mortato@idipaz.es (M.M.-M.); samara.palma@salud.madrid.org (S.P.-M.); cgcandela@salud.madrid.org (C.G.-C.); 2 Nutrition Research Group, La Paz University Hospital Institute for Health Research (IdiPAZ), 28046 Madrid, Spain; cristina.santurino@idipaz.es

**Keywords:** osteoporosis, bone mineral density, functional foods, nutrition, prevention

## Abstract

Osteoporosis is a multifactorial disease characterized by the loss of bone mass and deterioration of the internal structure of the bone, increasing the risk of fractures, and is becoming an economic and social problem. The main treatment is pharmacological, however, the population demands other therapies, such as foods with nutrients beneficial to bone health. Seventy-eight healthy menopausal women at risk of osteoporosis or untreated osteopenia were recruited for a randomized, parallel, double-blind clinical trial with two intervention groups: one group consumed a serving a day of the experimental enriched product (experimental group (EG)) and the other group (control group (CG)) consumed the same product without enrichment. The main objective was to compare the effect of consuming a dairy preparation to reconstitute, similar to yogurt when prepared, enriched in calcium, vitamin D, vitamin K, vitamin C, zinc, magnesium, L-leucine and probiotic (*Lactobacillus plantarum 3547*) on bone metabolism markers for 24 weeks. The EG showed a significantly increased bone mass compared to the CG (0.01 ± 0.03 vs. −0.01 ± 0.03 kg; *p <* 0.05). In addition, the EG maintained their bone mineral density (BMD) compared to the CG, whose BMD significantly decreased at the end of the study. For biochemical markers, the EG significantly increased the serum levels of the N-terminal propeptide of type I collagen (P1NP) bone formation marker (13.19 ± 25.17 vs. −4.21 ± 15.62 ng/mL; *p <* 0.05), and decreased the carbo-terminal telopeptide of type I collagen (CTx) bone resorption marker compared to the CG (−0.05 ± 0.19 vs. 0.04 ± 0.14 ng/mL; *p <* 0.05). On the other hand, the EG exhibited a significantly decreased systolic and diastolic blood pressure compared to the start of the study. Finally, the EG significantly increased their dietary calcium and vitamin D intake compared to the CG. In conclusion, the regular consumption of a dairy product to reconstitute enriched with bioactive nutrients improves bone health markers in menopausal women at risk of osteoporosis without pharmacological treatment.

## 1. Introduction

Osteoporosis is a skeletal disorder characterized by a loss of bone mass and a deterioration of the internal structure of bone [1]. It is a multifactorial disease that influences both genetic and physiological factors, such as age, sex, ethnicity or use of certain drugs, as well as lifestyle factors such as nutritional statuses, tobacco and alcohol consumption, inactivity, inadequate sun exposure or poor intake of bone-essential nutrients [2].

Damage to the bone resistance predisposes one to an increased risk of fracture, a fact that determines the morbidity-mortality that causes the disease. In this sense, menopausal and postmenopausal women are the demographic with the highest percentage of prevalence of osteoporosis, making up 30–50% of the world’s population with osteoporosis [3]. In Spain, despite being considered a country of moderate risk, the prevalence rate is the same as the global rate, with osteoporotic fracture rates of 15% and which double every seven years as age progresses [4].

Osteoporosis is therefore a major health problem, as it not only affects the health and quality of the life of sufferers, but also there are the negative impacts of the associated economic costs and the social problems of treatment. In 2010, the International Osteoporosis Foundation (IOF), together with the European Federation of Pharmaceutical Industry Associations (EFPIA), described the economic burden of the disease in the countries of the European Union and concluded that osteoporosis, as a whole (fractures, drug treatment and long-term care) costs 37 billion euros annually, and will increase by 25% in 2025 [5].

It is a disease that does not produce symptoms except when there is a fracture, therefore, an early diagnosis is essential [6]. An assessment of the risk requires the evaluation of the patient’s medical history and the identification of risk factors that may negatively affect bone health [7]. Next, the risk of fracture can be calculated using the Fracture Risk Assessment Tool (FRAX^®^), a validated tool created by the WHO that estimates the 10-year risk of developing an osteoporotic fracture [8]. Finally, to quantify the bone mineral density (BMD), defined as the amount of bone that exists once the growth period is complete [9], there are several techniques, but, with no doubt, the most commonly used is dual-energy X-ray absorptiometry (iDXA). This is a non-invasive technique which is fast to perform with low amounts of radiation and with very a high accuracy and reliability. In addition, iDXA provides the *T-score*, the marker needed to interpret the BMD and body composition.

In addition to radiological techniques, certain biochemical parameters related to phosphor-calcium metabolism can contribute to the diagnosis of osteoporosis by providing information on the rhythm of bone replacement. Among the markers of bone formation is the amino-terminal propeptide of pro-collagen type I (P1NP), and among the markers of bone resorption is the carbo-terminal telopeptide of type I collagen (CTX). The biochemical parameters derived from secondary osteoporosis, such as serum vitamin D and parathyroid hormone (PTH), among others, should also be assessed. 

On the other hand, it is a disease that, in many cases, could be prevented with an appropriate lifestyle, but, once established, the treatment of osteoporosis combines drugs, adapted physical activity and dietary measures, such as increasing the consumption of nutrient-rich foods essential for proper bone function. However, populations most often demand non-pharmacological treatments that help improve and prevent chronic diseases such as osteoporosis, and from this need functional foods arose. 

Functional foods are those that, in addition to being a source of energy and nutrients that ensure the survival of the individual, are able to promote health, improve well-being and reduce the risk of disease [10]. Currently, there are different types of matrices for functional foods, however, dairy foods are the most commonly used because they are beneficial for overall health and bone health, and because they allow the added substances to be stable [11].

Therefore, the main objective of the clinical trial was to evaluate the effect of regular consumption for 6 months of a dairy product enriched with bioactive nutrients (calcium, vitamin D, vitamin K, vitamin C, zinc, magnesium, L-leucine and probiotic *Lactobacillus plantarum 3547*) on bone metabolism markers in a group of healthy middle-aged women at risk of osteoporosis. 

## 2. Materials and Methods

### 2.1. Study Participants

For the present study, the Clinical Nutrition Department of La Paz University Hospital (HULP), Madrid (Spain) recruited and evaluated the participants. The inclusion criteria to be eligible for the study were as follows: menopausal women aged between 50 and 60 years; body mass index ≥18 kg/m^2^ and <35 kg/m^2^; osteopenia not being treated pharmacologically, or meeting two of the following criteria: <2 servings of dairy products per day and sedentary physical exercise measured with the International Physical Activity Questionnaire (IPAQ), or being smoker with >5 cigarettes a day. In addition, participants needed to agree to voluntarily participate in the study and sign the informed consent form. The exclusion criteria were as follows: women who have serious diseases (hepatic, renal, cancer), gastrointestinal diseases, mental illnesses or hyperthyroids/hypothyroids, antacid phosphate ligator treatments, glucocorticoid treatments, prior or concomitant treatment for bone metabolic diseases, hormone replacement therapy, who use soy isoflavones or nutritional supplements, women with a diet and/or pharmacological regimen for a decreased body weight in the 3 months prior to the start of the study, alcohol consumption >30 g/day ethanol, and women who use laxatives (>2 a week) and do not agree to eliminate them during the study.

All the participants gave their informed consent to take part in the study, which was approved by the Scientific Research and Ethics Committee of HULP (Code 4319) in accordance with the ethical standards of the Declaration of Helsinki [12]. The study is registered at http://clinical-trials.gov with number NCT02629341. 

### 2.2. Study Design

The study took the form of a randomized, parallel, double-blind clinical trial lasting 24 weeks. The participants (*n* = 78) were randomly assigned to 1 of 2 treatment groups: the experimental group (EG) or control group (CG). Patients from the EG consumed one serving a day of the experimental product enriched with bioactive bone nutrients, while patients from the CG consumed a daily ration of the same product but without enrichment. 

### 2.3. Experimental and Placebo Products

Both the experimental and placebo products were developed and labeled by the company CARINSA S.A., with its quality and safety controls. They also masked the products, making the presentation and characteristics of both products the same. The presentation of the product was made with a mix-pack, a container with two compartments separated by a membrane. In one compartment there was water (120 mL) and in the other the lyophilized product (30 g). When pressure was exerted on the vessel, the membrane would break and both ingredients bonded, giving a homogeneous, creamy, yogurt-like textured food. The composition of both products are shown in the Table 1.

### 2.4. Methods

#### 2.4.1. Diet

All participants received healthy eating guidelines but without increasing their intake of dairy and nutrients-rich foods in the study outside of their usual intake, so that it did not inflict bias on the study. The guidelines were reinforced by a nutritionist on all visits during the 6 months of the clinical trial and accession was verified with validated questionnaires (registration of 72 h and frequency of food consumption). The energy and nutritional content of the foods and beverages consumed were then calculated using DIAL software (Alce Ingeniería, Madrid, Spain).

At the end of study, a nutritionist gave specific healthy eating guidelines for the prevention of osteoporosis. 

#### 2.4.2. Anthropometric, Body Composition and Bone Mass Variables

Anthropometric measurements were taken at the beginning and end of the study using standard techniques, adhering to international norms set out by the WHO [13], by the dietitian researcher from the Nutrition Department of HULP. All measurements were made in the morning with the subject barefoot and wearing only underwear. The body composition and bone mass determined with dual-energy X-ray absorptiometry (iDXA (GE Healthcare, Madison, WI, USA)).

#### 2.4.3. Physical Activity 

At all trial visits it was recalled that the physical activity to be performed should be the usual one of each volunteer. The physical activity information for each attendant was collected through the International Physical Activity Questionnaire (IPAQ) [14] at the beginning of the intervention and at the end. At the end of study, the research staff gave guidelines for the prevention of osteoporosis. 

### 2.5. Biochemical Parameters

Two blood extractions were performed during the study (start and end), keeping the samples at 4–6 degrees until the analysis within a period of 48 h. Blood samples were taken after a 12 h overnight fast at the Extraction Unit of the Hospital Universitario La Paz (Madrid, Spain). A biochemical serum lipid profile (HDL-cholesterol and LDL-cholesterol and triglycerides) were determined using an ADVIA^®^ 1200 Siemens Healthcare Diagnostics Inc (Tarrytown, NY, USA) and glucose and insulin determinations were performed by an enzymatic-spectrophotometric assay using an ADVIA Centaur^®^ Immunoassay System (Tarrytown, NY, USA). Safety biomarkers (alanine amino transferase, aspartate amino transferase, alkaline phosphatase, bilirubin and creatinine concentrations) and some bone metabolism markers (calcium, phosphorus, vitamin D, PTH) were determined on the blood using the enzyme-spectrophotometric method using the Olympus Diagnostic AU 5400 automatic analyzer (ClearChem Diagnostic, Ontario, CA, USA). For measurements of the P1NP and CTx parameter, the “ECLIA” electrochemoluminescence immunoassay kit was used, which determines the amino-terminal and carbo-terminal end present in the blood.

### 2.6. Adverse Events

Adverse events were documented in all trial visits. An adverse event was defined as any unfavorable, unintended effect. All such events were recorded along with the symptoms involved (bad breath, nausea, vomiting, diarrhoea, constipation, others). An unexpected adverse event was related to the consumption of the product if the causality was highly probable, probable, possible, or conditioned. The intensity was defined in three graduations: mild (momentary and tolerated by the subject), moderate (interferes with the normal activity of the subject) or severe (disables the normal activity of the subject).

### 2.7. Statistical Analysis 

The sample size was calculated with nQuery Advisor Release 2.0 (Stadistical Solutions, Boston, MA) based on vitamin D levels as a predictor variable according to Hoeck et al. [15], with a confidence level of 95% (power 0.095), precision of 5% (alpha error of 0.05) and taking into account a drop out of 20% (*n* = 78). Descriptive statistics were calculated (the mean and standard deviation for quantitative variables and percentages for qualitative variables). All variables were tested for the distribution of normality and uniformity of the variance, using a Kologorov–Smirnov test (or Shapiro–Wilk if necessary) with a Levene’s test, respectively. The possible association between variables was analyzed through the chi-squared test followed by Fisher’s exact test (qualitative variables such as gender) or by a t-test (continuous variables) applying the continuity correction. 

When the distribution of the results was homogeneous (parametric variables), the degree of significance of the differences in the means was determined by a Student’s t test for related samples when it came to paired variables (intragroup: from week 0 to week 24), and for independent samples (week 0 CG vs. week 0 EG; change CG vs. change EG) when they were unpaired variables. When the distribution of the results was not homogeneous (non-parametric variables), the degree of significance was determined by the Wilcoxon test for dented samples (when it came to paired variables) and Mann-Whitney U test for independent samples (when it came to unpaired variables). 

A *p* value of <0.05 was determined to indicate significance. All analyses were performed using SPSS v.15.0 software (SPSS Inc., Chicago, IL, USA). 

## 3. Results

### 3.1. Recruitment and Study Population

**Figure 1:**
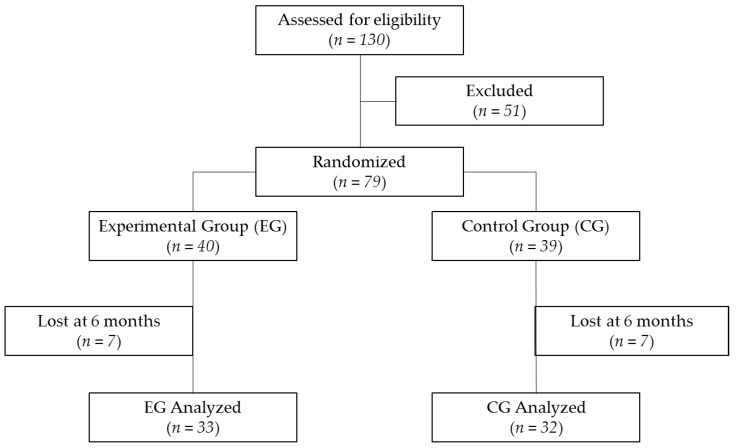
Flow chart describing the present trial.

The study was performed between January and July 2015. A total of 79 women were eligible for inclusion. Some 14 participants were lost at 6 months (seven in the EG and seven in the CG); thus 65 participants completed the 24-week study (Figure 1). These withdrawals were not related to the consumption of the study’s dairy products, but for other reasons like study length fatigue, disinterest, lack of the consumption product, diagnosis of disease, and an inability to contact to arrange the visits.

### 3.2. Baseline Characteristic, Physical Activity and Blood Pressure Variables

The average age of the population studied was 56 ± 3.61 years (ages from 52 to 60 years), with 11% being active smokers (eight cigarettes a day, on average) with no significant differences between groups.

The evaluated population showed a low level of physical activity, with a sedentary rate of 44% and very mild physical activity at 34%, remaining stable at the end of the study. No statistically significant differences were found between treatment groups.

As for blood pressure, no statistically significant differences were found between the two treatment groups, staying within normal levels for the general population (120/80 mmHg). However, at the end of the intervention, the EG observed a significant statistical decrease in systolic blood pressure (SBP) and diastolic blood pressure (DBP), with respect to the start of the study (SBP 112.85 ± 17.27 vs. 104.12 ± 10.62 mmHg; *p* < 0.05; DBP 77.12 ± 10.59 vs. 71.79 ± 10.83 mmHg; *p* < 0.01).

### 3.3. Adverse Events

No adverse events related to the consumption of the dairy products were documented throughout the clinical trial. Only one participant from the experimental group attributed constipation problems to the consumption of the enriched dairy product, whose causality was conditioned and the intensity defined as mild. After a few days, these symptoms disappeared and the participant continued to the end of the intervention. Biochemical safety parameters did not show significant changes between the treatment groups (Table 2). A significant increase in glucose between week 0 and week 24 was observed in the experimental group (from 90.76 ± 7.96 to 94.91 ± 8.77 mg/dL; *p* < 0.01) as well as in the control group (from 92.13 ± 7.43 to 96.13 ± 8.30 mg/dL; *p* < 0.01). This increase, however, was not clinically relevant since the means of glucose were within the parameters of normality for the sex and age of the population. The total cholesterol and LDL cholesterol were reduced from the beginning to the end of the intervention, however they did not show significant differences between treatment groups (−8.53 ± 18.5 vs. −7.12 ± 19.91 mg/dL). The rest of the biochemical safety parameters remained stable throughout the intervention.

### 3.4. Dietetic Intake Variable

After the end of the clinical trial, some of the dietary parameters evaluated showed significant changes (Table 3) although the trial did not include dietary intervention. Despite this, it was recommended to follow a healthy diet, without increasing food consumption that could interfere with the study product. Dietary guidelines have been possibly responsible for the changes observed in both groups at the end of study, as a decrease in the total caloric intake, a lower carbohydrate intake, an improvement in dietary lipid profiles, decreased dietary cholesterol and increased dietary fiber consumption.

It was observed that the dietary intake calcium (35.28 ± 27.21 vs. 183.36 ± 334.05 mg/day; *p* < 0.05) and vitamin D (0.40 ± 3.31 vs. 2.75 ± 3.19 µg/day; *p* < 0.05) increased significantly in the EG at the end of the study compared to the CG (Table 1). The increase in dietary calcium in the EG meant that the percentage of women who reached at least 1000 mg daily, as recommended by clinical guides, increased from 12% to 42% after the intervention, while the CG continued with the same 10% population as at start of the study.

Regarding vitamin D intake, although the mean EG value was higher than the initial, it is important to note that in both groups the intake remains poor (<10 µg/day (<800 UI/day)).

### 3.5. Anthropometric, Bone Body Composition Variables

The mean values of the anthropometric parameters and body composition are outlined in Table 4. In both groups it was observed that their body mass indexes (BMIs) correspond to overweight and remains stable at the end of the clinical trial without significant differences between groups. Equally the waist circumference, in both groups, exceeded the recommended level of health for the European women (>80 cm).

Regarding the bone body composition, both groups had a similar bone masses, BMDs and T-scores at the start of the study without differences, however, at the end of the intervention, the EG increased its bone mass significantly compared to the CG (−0.01 ± 0.03 vs. 0.01 ± 0.03 kg; *p* < 0.05). In fact, the BMD remained stable while in the CG it declined significantly from baseline (0.00 ± 0.00 vs. 0.01 ± 0.01 kg/cm²; *p* < 0.05). In addition, 28% of the CG had osteopenia at the start of the study, and at the end this increased to 31%, while in the EG the percentage of osteopenia decreased significantly at the end of the intervention (from 30% to 24%).

### 3.6. Biochemical Parameters

Table 5 shows the results obtained from the main biochemical parameters of bone metabolism. Both P1NP and CTx are good markers of bone metabolism by relating directly to the BMD. After the completion of the intervention, the improvement of both parameters in women of the EG was observed (P1NP: 13.19 ± 25.17 vs. −4.21 ± 15.62 ng/mL; *p* < 0.05; CTx: −0.05 ± 0.19 vs. 0.04 ± 0.14 ng/mL; *p* < 0.05).

As for the hormones regulating phosphor–calcium metabolism, a clear decrease in the PTH and an increase in serum vitamin D in the EG, with respect to CG, are observed (4.49 ± 13.94 vs. 1.93 ± 6.60 ng/mL; *p* < 0.05). In fact, the increase in the latter meant that 15% of women who belonged to this group reached the sufficiency ranges (>30 ng/mL), as all the volunteers in the study had hypovitaminosis D at the beginning.

## 4. Discussion

Osteoporosis is a very prevalent chronic disease that represents a major public health problem worldwide because of its association with fractures and fragility [16]. With such a high economic cost in a country were expectancy is increasing, especially in the female sex, it is necessary to look for alternatives for both treatment and prevention which are not pharmacological and, in turn, reduce cost. Hence, the diet in these processes can be understood not only as a biological resource, but as a beneficial treatment. For this reason, this study assessed the consumption of a dairy product to reconstitute enriched with bioactive nutrients on the bone metabolism markers in a group of healthy middle-aged women at risk of osteoporosis.

Regular consumption of the enriched dairy product did not present adverse events and, as in other studies that used *Lactobacillus*, its consumption was not associated with changes in the biochemical safety parameters [17].

High blood pressures in women at risk of osteoporosis are closely related to the condition. Since the 1990s, multiple epidemiological studies have associated an elevated blood pressure with a low bone density [18], and in women, a large prospective study established a positive association between blood pressure and BMD loss, with a significant increase in femoral BMD bone turnover in those with high baseline blood pressure figures [19]. In this sense, no differences were found in the studied population and blood pressures were within normal ranges. However, at the end of the intervention, women who consumed of the enriched dairy product had a decrease in their SPB and DPB compared to the start of the study. This may be due, first of all, to the basic ingredients of the dairy product. There are several clinical trials, systematic reviews and meta-analyses that show the positive effect of dairy ingredients (proteins and minerals) on blood pressure [20,21,22]. Feket et al. conducted a randomized, double-blind, placebo-controlled clinical trial, in which milk proteins were provided for 8 weeks to healthy adult men and women, achieving a significant reduction in their SPBs and DPBs [23]. In fact, other clinical trials conducted with dairy peptides in mildly hypertensive subjects have been shown to significantly reduce blood pressure in these individuals [24].

In addition, the results obtained may be related to the calcium enrichment of the dairy preparation to reconstitute, since this mineral is involved in the mechanism of blood pressure modulating peripheral vascular resistance. One of the most recent studies is the randomized and controlled clinical trial of Billington et al., in which a calcium supplement (1000 mg of calcium citrate) and a placebo was administered to postmenopausal women with the aim of evaluating the changes in blood pressure. Blood pressure was recorded at 2 h, 4 h and 6 h after calcium or placebo ingestion and found that both the SBP and DBP decreased significantly (−6 and −9 mmHg, respectively). The authors concluded that the use of calcium supplements in postmenopausal women could help reduce high blood pressure [25]. However, a few years ago, several research groups reported on the harmful effects of using calcium supplements with regards to vascular events in healthy postmenopausal women with a low calcium intake or osteoporosis [26,27,28], and despite intervention studies promoting its consumption [29], the use of calcium supplements in this population has been questioned on an ongoing basis [30]. Given the controversy and knowing that calcium consumption in adequate amounts in women can help prevent bone loss and the risk of fractures [31], it is increasingly preferred to use foods with a significant calcium content [32,33], like the one used in this study. In fact, the increased intake of calcium and dietary vitamin D in women is likely to result from the consumption of the dairy product to reconstitute, as these results are based on clinical trials that use fortified foods to increase the intake of these nutrients [34,35,36].

On the other hand, the anthropometry characteristics remained stable at the end of the intervention in all populations, since the design of the study did not have dietary interventions that could modify these parameters. Acquiring information on the body composition of postmenopausal women is essential because it is known that the time elapsed since the onset of menopause alters the parameters of the body compartments interfering with BMD [37]. For this reason, the iDXA, a radiological method that allows a more accurate and reliable method for determining the amount of bone mass [38], was used. In the studied population, it has been observed that women who consume the enriched dairy product presented an increase in bone mass. Considering these clinically relevant results, it should be kept in mind that the participants were menopausal women who were not receiving pharmacological treatments and who were at risk of osteoporosis—some of the women already had osteopenia. The bone compartment is quantified through the BMD, which informs the amount of bone that exists after the growth period is complete [9]. However, a disadvantage of the method used to assess the bone mass is that it is not significantly sensitive to predict fractures [39]. In relation, the T-score is the parameter that allows the BMD to be interpreted and a diagnosis is made from the bone content. In the present clinical trial, both the BMD and T-score, have behaved differently. In this sense, women who consumed the enriched dairy product had both parameters remain stable, in contrast with the women of the control group whose parameters depleted. Since the intervention was designed for this purpose, it is possible to attribute these improvements to regular consumption for 24 weeks of the dairy product. In fact, several studies on postmenopausal women evaluate the use of calcium and vitamin D enriched dairy products on BMD that resemble the results obtained in this clinical trial. One of them is the one conducted by Cleghorn et al. These researchers conducted a 2-year randomized study in 115 postmenopausal women without hormone treatment or other therapies that may affect the bone, and who had calcium consumptions of less than 1000 mg/day. Participants were randomly assigned to two groups, one receiving a 3-litre supplement of calcium-fortified milk and another that followed their usual diet during the first years. After that year and during the second year, it was the second group that received the enriched supplement and the first group who returned to their usual diet. At the end of the study, bone loss was significantly lessened in women who took the milk supplement when they were on their usual diet [40]. Additionally, Chen et al. conducted a randomized, double-blind, placebo-controlled, 2-year-long clinical study to examine the effect of a calcium and vitamin D enriched milk powder on the bone of 210 menopausal women (aged 50–60 years). After the intervention ended, the women who consumed the enriched milk were the ones who had a significantly improved BMD compared to the placebo group [41].

In addition to product enrichment, the matrix used should be highlighted. In fact, the study by Gui et al. reveals that it is cow’s milk, rich in phosphorus, milk proteins, magnesium, among others, and enriched in calcium, which has effects on BMD. To reach this conclusion, these researchers conducted a randomized, open and controlled clinical trial, in 141 menopausal women (aged 45–65 years). Participants were assigned to three different treatment groups: (A) consumption of 250 mL of milk added with 250 mg of calcium per day; (B) consumption of 250 mL of a soy drink added with 250 mg of calcium per day; (C) no milk consumption. During the 18 months of the study, the BMD of the spine and hip was evaluated at baseline, 6, 12 and 18 months. Once the intervention was complete, statistically significant differences were observed between enriched cow’s milk and the other two treatment groups. In fact, women in group A had a significantly increased hip and femur BMD compared to the study’s start, while group *B* and *C* had a decrease in these and other bone structures [42].

On the other hand, both the bone formation marker (P1NP) and the bone resorption marker (CTx) improved significantly with the consumption of the enriched dairy product. In this sense, there are other clinical studies that have used a dairy food enriched with calcium and vitamin D that demonstrate the effect of these on bone metabolism in women of different ages. One of them is the randomized, double-blind and controlled clinical study conducted by Kruger et al. on 121 menopausal women (aged 55–63 years), with the aim of evaluating markers of bone health. Participants were randomized into two intervention groups. The control group (*n* = 60) consumed a normal milk that provided 428 mg of calcium per day, and the intervention group consumed a milk enriched in 12,000 mg of calcium, 15 µg vitamin D, zinc, magnesium and other micronutrients. After 52 weeks, the intervention demonstrated a significant suppression of CTx and a significant increase in the P1NP levels of the groups that consumed enriched milk [43]. Similarly, these same researchers conducted another study with women of different ages who were give a milk powder enriched in 1000 mg of calcium or a supplement with 1000 mg of calcium gluconate/carbonate salt. Following the analysis, they concluded that the response of CTx and P1NP was significantly higher when fortified milk was consumed, concluding that a fortified milk matrix brings more benefits to bone metabolism than isolated supplementation [44].

Furthermore, the dairy preparation, in addition to being enriched in calcium, vitamin D and other micronutrients, had a probiotic that has been able to favor the results obtained in terms of BMD and biochemical parameters. In fact, the clinical trial using postmenopausal women of Jafarnejad et al., who took a bacteria supplement with 500 mg of calcium and 200 UI of vitamin D, observed that the P1NP increased and CTx decreased significantly compared to those who had a placebo [45], and the recent reviews of Rizzoli and Biver in 2018 and 2020 showed how the consumption of fermented dairy products, such as yogurt, positively influences bone metabolism [46] and it attenuates age-related bone loss [47,48].

As for serum vitamin D levels, it should be remembered that there is an epidemic of hypovitaminosis D in menopausal women, and, in fact, all women in the study had low levels at the start of the study. Therefore, the increase in levels only in the experimental group may be due to the enriched dairy preparation. This is shown in other studies [49,50] that support these results, such as the clinical trial conducted by Manios et al. In this study, 79 postmenopausal women were divided into two groups. The control group consumed their usual diet alongside an enriched dairy product, and the intervention group retained their usual diet plus a dairy product enriched with 5.7 g of vitamin D. After 8 weeks of intervention in the winter months, the treatment group had significantly increased serum vitamin levels relative to the control group [51]. Similarly, Krausti et al. investigated the bioavailability of vitamin D in enriched milks compared to individual supplements and demonstrated that absorption is greater when used by these foods, emphasizing the value of using dairy products [52].

Finally, calcium homeostasis is also regulated by calcitonin and PTH. The first is a hormone that activates quickly when there is an increase in the ion concentration in calcium and, over a few minutes, lowers this elevation by favoring the mineral deposits. Despite its important function on bone metabolism, no significant differences between the groups were observed after the completion of the study. These results could be explained by the physiological state of menopause being associated with a dietary calcium intake. In other words, when there is a low calcium intake, such as in the study population and which is very common in menopausal and postmenopausal women, PTH is activated to release calcium from the bone and thus maintain plasmatic levels. An elevation in the blood concentration of active calcium to calcitonin is able to decrease them, but being the most potent PTH, the effect of calcitonin is partly nullified, reducing its circulating levels [53]. As for PTH, the average levels in both of the treatment groups were above normal values (10–55 pg/mL); this may be due to a poor calcium intake, vitamin D deficiency or estrogen deficiency [54]. At the end of the intervention, although the levels remained higher than normal values, women from the experimental group showed a decrease which may be due to their consumption of the enriched dairy preparation. Thus, these results are supported by other studies with similar results. A study conducted by Tenta et al. gave 40 postmenopausal women a dairy enriched product with 1200 mg of calcium and 22.5 µg of vitamin D, compared to women with a regular diet (control group) and, after 30 months of intervention, a significant increase in PTH levels was observed in the control group while concentrations of this hormone decreased in the intervention group [31]. Similarly, a study conducted by Bonjour et al. in 56 postmenopausal women also achieved similar results [55].

Among the limitations of this study is the lack of regulated physical activity, the benefits on bone health of which are widely documented [56,57]. In this sense, it is possible that regular monitored physical exercise could provide improvements on bone biomarkers in both treatment groups. Another disadvantage of the clinical trial is the limited number of bone biomarkers evaluated. Additionally, the period of intervention could be considered relatively short, however, it was enough to observe metabolic changes on bone health. Therefore, the development of clinical trials carried out for longer periods of time and with a greater number of parameters related to bone health are desirable.

According to these evidences, as PTH levels have decreased significantly in the EG, the fortification of dairy products could provide a greater advantage against accelerated bone resorption in women at risk of osteoporosis, compared to equivalent non-fortified foods [58].

## 5. Conclusions

The study shows that the consumption of an experimental dairy product resulted in a significant improvement in bone mass content and managed to mitigate the loss of bone mineral density, unlike in the control group. In addition, women who consumed the dairy product had an improvement in their plasma levels in bone metabolism markers compared to the control group. Similarly, thanks to the consumption of the dairy product, the intake of calcium and vitamin D improved significantly. Overall, the consumption of a dairy product to reconstitute enriched with bioactive nutrients for bone over 6 months improved the bone health markers in healthy middle-aged menopausal women who were at risk of osteoporosis without pharmacological treatment.

As a result, the consumption of functional foods with a milk matrix enriched in nutrients essential for bone health could contribute to the primary prevention of osteoporosis and thus decrease the morbid mortality rates and the costs associated with the disease.

## Figures and Tables

**Table 1:** Nutritional composition of products (150 g).

	EG	CG
Energy (kcal)	126	123
Lipid (g)	2.06	2.06
saturated (g)	1.8	1.8
Carbohydrates (g)	23	23
sugars (g)	18	18
Protein (g)	1.8	1.8
Calcium (mg)	501	227
Vitamin D3 (μg)	6	0.015
Vitamin K (μg)	80	-
Vitamin C (mg)	100	-
Zinc Gluconate (mg)	10.39	-
Dicitrate of Trimagnesium (mg)	250.5	-
L-Leucin (g)	1	-
*Lactobacillus plantarum 3547* (ufc)	1 × 10^10^	-

**Table 2:** Biochemical safety parameters of the study’s participants before and after the intervention.

		CG			EG		
		Week 0	Week 24	Change	Week 0	Week 24	Change
Fasting plasma glucose	mg/dL	92.13 ± 7.43	96.13 ± 8.3 *	4.00 ± 6.74	90.76 ± 7.96	94.91 ± 8.77 *	4.15 ± 8.78
Plasma insulin	µU/mL	8.78 ± 5.38	9.88 ± 8.11	1.10 ± 6.38	8.50 ± 4.82	9.42 ± 4.23	0.91 ± 3.79
Total cholesterol	mg/dL	217.26 ± 29.69	210.59 ± 24.95 *	−6.69 ± 23.3	211.18 ± 35.41	204.88 ± 35.41 *	−6.30 ± 22.09
LDL-cholesterol	mg/dL	137.09 ± 27.33	128.56 ± 23.00 *	−8.53 ± 18.5	131.03 ± 29.87	123.91 ± 26.93 *	−7.12 ± 19.91
HDL-cholesterol	mg/dL	63.19 ± 14.71	62.16 ± 12.54	−1.03 ± 10.3	61.91 ± 14.16	61.71 ± 13.63	−0.12 ± 5.73
Triglycerides	mg/dL	84.84 ± 31.12	87.09 ± 46.26	2.25 ± 31.85	91.76 ± 43.68	94.30 ± 39.44	2.54 ± 27.99
Creatinine	mg/dL	0.64 ± 0.10	0.65 ± 0.12	0.01 ± 0.02	0.64 ± 0.09	0.65 ± 0.08	0.01 ± 0.01
Urate	mg/dL	4.32 ± 1.17	4.37 ± 1.13	0.05 ± 0.92	4.13 ± 0.82	4.18 ± 0.98	0.05 ± 0.58
Aspartate transaminase	UI/L	22.81 ± 7.00	22.47 ± 6.34	−0.34 ± 5.26	24.33 ± 8.24	24.52 ± 11.24	0.18 ± 5.97
Alanine transaminase	UI/L	23.13 ± 10.45	23.13 ± 11.86	0.00 ± 5.35	24.12 ± 10.74	22.70 ± 11.94	−1.42 ± 8.12
Alkaline phosphatase	UI/L	87.75 ± 23.26	88.05 ± 23.64	0.30 ± 11.43	81.42 ± 19.53	81.75 ± 17.69	0.33 ± 10.6
Bilirubin	mg/dL	0.53 ± 0.16	0.57 ± 0.15	0.04 ± 0.21	0.55 ± 0.22	0.59 ± 0.19	0.04 ± 0.21

Data are expressed as the means ± SDs. Difference from the start of the intervention (* *p* < 0.05).

**Table 3:** Dietetic intake of the study participants before and after the intervention.

		CG			EG		
		Week 0	Week 24	Change	Week 0	Week 24	Change
Energy	(kcal)	2025.24 ± 238.45	1829.4 ± 355.5 **	−195.76 ± 359.9	2036.14 ± 244.83	1818.7 ± 304.1 **	−217.39 ± 306.7
Carbohydrates	(%)	36.13 ± 6.15	33.35 ± 6.38 **	−2.78 ± 6.16	36.46 ± 5.94	32.89 ± 7.24 **	−3.57 ± 6.38
Proteins	(%)	16.94 ± 3.00	17.78 ± 3.05	0.84 ± 2.31	18.15 ± 2.53	17.89 ± 3.08	−0.27 ± 3.42
Lipid	(%)	46.93 ± 3.61	48.87 ± 3.52 *	1.94 ± 3.32	45.39 ± 4.46	48.22 ± 8.25 *	2.83 ± 2.29
SFA	(%)	14.97 ± 3.32	15.39 ± 2.21	0.42 ± 1.08	14.57 ± 2.48	15.26 ± 2.57	0.69 ± 2.27
MFA	(%)	26.26 ± 4.10	27.70 ± 4.60 *	1.44 ± 5.79	24.89 ± 4.50	27.54 ± 5.68 *	2.65 ± 5.51
PFA	(%)	5.70 ± 1.90	5.78 ± 1.35	1.08 ± 2.30	5.93 ± 2.23	6.42 ± 2.64	0.49 ± 2.61
Total Cholesterol	(mg/day)	324.38 ± 70.62	291.48 ± 92.94 *	−32.90 ± 90.93	339.46 ± 92.16	289.43 ± 80.80 *	−50.03 ± 19.02
Fibre	(g/day)	22.30 ± 7.62	26.01 ± 5.17 *	3.71 ± 13.71	21.42 ± 5.69	29.73 ± 5.51 *	8.31 ± 36.52
Calcium	(mg/day)	759.24 ± 13.74	794.52 ± 18.62	35.28 ± 27.21	776.39 ± 29.67	959.75 ± 266.2 *	183.36 ± 334.0 #
Vitamin D	(µg/day)	12.99 ± 2.34	13.39 ± 2.59	0.40 ± 3.31	13.35 ± 2.61	16.10 ± 2.93 *	2.75 ± 3.19 #

Data are expressed as the means ± SDs. SFA: saturated fatty acids; MFA: monounsaturated fatty acids; PFA: polyunsaturated fatty acids. Difference from the start of the intervention (* *p* < 0.05; ** *p* < 0.01). The change at the end of the intervention was significantly different from the control group (# *p* < 0.05).

**Table 4:** Anthropometric parameters and body composition of the study participants before and after the intervention.

		CG			EG		
		Week 0	Week 24	Change	Week 0	Week 24	Change
Weight	(kg)	66.71 ± 12.32	66.21 ± 12.10	−0.49 ± 2.93	67.22 ± 10.13	66.61 ± 9.41	−0.61 ± 3.52
BMI	(kg/m^2^)	26.47 ± 4.11	26.39 ± 4.20	−0.08 ± 1.13	26.34 ± 3.19	26.2 ± 3.20	−0.14 ± 1.53
Waist circ.	(cm)	88.89 ± 11.54	88.7 ± 12.22	0.19 ± 7.02	89.01 ± 11.42	88.54 ± 11.81	−0.47 ± 5.82
BMD	(kg/cm^2^)	1.05 ± 0.10	1.04 ± 0.10 **	−0.01 ± 0.01	1.04 ± 0.11	1.04 ± 0.11	0.00 ± 0.00 *#*
T-Score		−0.28 ± 1.04	−0.36 ± 0.10 **	−0.08 ± 0.13	−0.40 ± 1.04	−0.40 ± 1.04	0.00 ± 0.00 *#*
Z-Score		0.36 ± 0.94	0.37 ± 0.90	0.02 ± 0.22	0.20 ± 1.04	0.23 ± 1.03	0.03 ± 12
BM	(kg)	2.09 ± 3.12	2.08 ± 0.30 *	−0.01 ± 0.03	2.07 ± 0.26	2.08 ± 0.26 *	0.01 ± 0.03 *#*
FM	(kg)	27.32 ± 9.30	27.24 ± 8.84	−0.08 ± 0.02	27.52 ± 7.53	27.78 ± 7.57	0.26 ± 1.13
LM	(kg)	36.61 ± 3.76	36.23 ± 3.98	−0.38 ± 0.22	37.69 ± 3.59	37.53 ± 3.61	−0.17 ± 0.86
FFM	(kg)	38.71 ± 3.98	38.31 ± 4.17	−0.40 ± 0.19	39.76 ± 3.72	39.59 ± 3.75	−0.17 ± 0.86

Data are expressed as the means ± SDs. BMI: body mass index; waist circ.: waist circumference; BMD: bone mineral density; BM: bone mass; FM: fat mass; LM: lean mass; FFM: fat free mass. Difference from the start of the intervention (* p < 0.05; ** p < 0.01). The change at the end of the intervention was significantly different from the control group (# *p* < 0.05).

**Table 5:** Biochemical parameters of bone metabolism of the study participants before and after the intervention.

		CG			EG		
		Week 0	Week 24	Change	Week 0	Week 24	Change
P1NP	ng/mL	59.37 ± 13.70	55.16 ± 17.15	−4.21 ± 15.62	58.17 ± 13.26	71.36 ± 5.50 **	13.19 ± 25.17 #
CTx	ng/mL	0.49 ± 0.20	0.53 ± 0.23	0.04 ± 0.14	0.50 ± 0.19	0.46 ± 0.16	−0.05 ± 0.19 #
Calcium	mg/dL	9.65 ± 0.29	9.62 ± 0.26	−0.03 ± 0.33	9.54 ± 0.35	9.57 ± 0.30	0.03 ± 0.38
Phosphorus	mg/dL	3.63 ± 0.52	3.68 ± 0.39	0.05 ± 0.46	3.69 ± 0.46	3.77 ± 0.34	0.08 ± 0.41
Vitamin D	ng/mL	17.97 ± 9.70	19.89 ± 5.13	1.93 ± 6.60	19.24 ± 7.07	23.73 ± 15.6 *	4.49 ± 13.94 #
Calcitonin	pg/mL	2.09 ± 0.40	2.07 ± 0.31	−0.02 ± 0.28	2.12 ± 0.43	2.13 ± 0.01	0.01 ± 0.43
PTH	pg/mL	83.19 ± 26.63	100.47 ± 14.2 *	17.28 ± 12.46	85.89 ± 24.35	82.12 ± 21.06	−3.77 ± 23.29 #

Data are expressed as the means ± SDs. P1NP: amino-terminal propeptide of pro-collagen type I; CTx: carbo-terminal telopeptide of type I collagen; PTH: parathyroid hormone. Difference from the start of the intervention (* *p* < 0.05; ** *p* < 0.01). The change at the end of the intervention was significantly different from the control group (# *p* < 0.05).
